# Entropy Analysis of Electroencephalography for Post-Stroke Dysphagia Assessment

**DOI:** 10.3390/e27080818

**Published:** 2025-07-31

**Authors:** Adrian Velasco-Hernandez, Javier Imaz-Higuera, Jose Luis Martinez-de-Juan, Yiyao Ye-Lin, Javier Garcia-Casado, Marta Gutierrez-Delgado, Jenny Prieto-House, Gemma Mas-Sese, Araceli Belda-Calabuig, Gema Prats-Boluda

**Affiliations:** 1 Centro de Investigación e Innovación en Biotecnología (Ci2B), Universitat Politècnica de València (UPV), 46022 Valencia, Spain; avelher@upv.edu.es (A.V.-H.); jimahig@etsii.upv.es (J.I.-H.); jlmartinez@eln.upv.es (J.L.M.-d.-J.); yiye@ci2b.upv.es (Y.Y.-L.); jgarciac@ci2b.upv.es (J.G.-C.); 2 Medical Rehabilitation Department, Medium- and Long-Term Care of Hospital Pare Jofré, 46017 Valencia, Spain; 3 Medical Rehabilitation Department, Hospital La Pedrera, 03700 Alicante, Spainbelda_marcala@gva.es (A.B.-C.)

**Keywords:** dysphagia, stroke, electroencephalogram, signal processing, entropy

## Abstract

Affecting over 50% of stroke patients, dysphagia is still challenging to diagnose and manage due to its complex multifactorial nature and can be the result of disruptions in the coordination of cortical and subcortical neural activity as reflected in electroencephalographic (EEG) signal patterns. Sample Entropy (SampEn), a signal complexity or predictability measure, could serve as a tool to identify any abnormalities associated with dysphagia. The present study aimed to identify quantitative dysphagia biomarkers using SampEn from EEG recordings in post-stroke patients. Sample entropy was calculated in the theta, alpha, and beta bands of EEG recordings in a repetitive swallowing task performed by three groups: 22 stroke patients without dysphagia (controls), 36 stroke patients with dysphagia, and 21 healthy age-matched individuals. Post-stroke patients, both with and without dysphagia, exhibited significant differences in SampEn compared to healthy subjects in the alpha and theta bands, suggesting widespread alterations in brain dynamics. These changes likely reflect impairments in sensorimotor integration and cognitive control mechanisms essential for effective swallowing. A significant cluster was identified in the left parietal region during swallowing in the beta band, where dysphagic patients showed higher entropy compared to healthy individuals and controls. This finding suggests altered neural dynamics in a region crucial for sensorimotor integration, potentially reflecting disrupted cortical coordination associated with dysphagia. The precise quantification of these neurophysiological alterations offers a robust and objective biomarker for diagnosing neurogenic dysphagia and monitoring therapeutic interventions by means of EEG, a non-invasive and cost-efficient technique.

## 1. Introduction

### 1.1. Dysphagia

Dysphagia, or swallowing disorder, is the difficult passage of a food bolus through all the swallowing phases [1], and it can involve physiological complications including difficulty swallowing, dehydration and nutrient deficiency, and choking or pneumonia due to regurgitated food entering the respiratory tract [1]. This swallowing dysfunction can be oropharyngeal or esophageal; oropharyngeal dysphagia is caused by the delay of the liquid or solid bolus in entering the oropharyngeal phase of swallowing and difficulty in bringing the bolus into the neck of the esophagus [2], with 80% of cases of oropharyngeal dysphagia mainly being caused by a neuromuscular problem [3].

Approximately 14% to 18% of hospitalized patients suffer from dysphagia, while between 30% and 60% of patients in nursing homes report symptomatic dysphagia [2]. Oropharyngeal dysphagia is quite common and occurs in up to 50% of the elderly and up to 50% of individuals with neurological involvement, while most of these go undiagnosed and receive no treatment [4]. The main cause of dysphagia in neurodegenerative diseases is Parkinson’s disease, in which 50% of the patients show oropharyngeal symptoms [4]. Dysphagia affects more than 50% of stroke survivors, with approximately 12% of patients suffering from this disorder 6 months after the stroke [5]. A systematic review of the costs of dysphagic patients in the USA reported that the annual cost of caring for these patients is USD 12,715, somewhat higher than other groups (40.36% per annum) [6]. A significant increase in costs was also reported for patients with oropharyngeal dysphagia [6].

### 1.2. Actual Diagnosis and Treatment of Dysphagia

Diagnosing dysphagia is challenging because of the complexity of swallowing and its multifactorial causes, including neurological, structural, and functional disorders, often requiring specialized multidisciplinary evaluation. The gold standard techniques include videofluroscopy (VFS) and fibroscopic swallowing evaluation (FEES), while the usual method is volume–viscosity clinical examination (V-VST) [7,8]. VFS obtains detailed images of swallowing dynamics using X-rays and contrast, although its drawbacks include radiation exposure, high costs, the need for specialized equipment, and subjective expert evaluation [7,8]. FEES involves the direct visualization of the pharyngeal structures by a flexible endoscope and is useful for detecting silent aspiration and anatomical abnormalities. However, it cannot visualize the oral swallowing phase and patient discomfort [7,8]. V-VST is the most widely used method in clinical practice for assessing oropharyngeal dysphagia. The procedure requires the patient to ingest boluses ranging from 5 to 20 mL with viscosity adjustments based on the patient’s swallowing difficulty, including the consistencies of water, nectar, and puddings [7], but relies heavily on the evaluator’s expertise and does not provide visual anatomical information, making this test highly subjective and somewhat deficient in diagnosing dysphagia both quantitatively and objectively.

Current treatments, including neurorehabilitation, diet modification, percutaneous endoscopic gastrostomy, and surgical interventions, focus on minimizing the risk of aspiration pneumonia and ensuring the patient’s safety [9]. No standardized tool is currently available for evaluating the progression of dysphagia or the effectiveness of therapeutic interventions, which hinders the patient’s recovery process and delays clinical decisions while ensuring safety. Ultimately, a standardized approach is essential for the comprehensive management of the disorder.

### 1.3. Entropy in EEG Signals

In the literature, swallowing event-related desynchronization (ERD) in beta band power has been found in the motor cortex, which may be associated with the activation of neural circuits involved in motor control [10] which reveals that the brain is actively preparing or coordinating the motor activity needed for swallowing. While ERD has proven to be a valuable source of information, the development of a comprehensive diagnostic tool remains elusive due to the variability in response observed between subjects and epochs, so the scientific community is actively seeking novel quantitative biomarkers that could offer a more reliable dysphagia diagnosis.

Many studies have shown that swallowing is primarily regulated by the cerebral cortex, requiring the coordination of both cortical and subcortical neural activity [11,12,13,14,15], which may be reflected in the degree of organization of EEG signals. Swallowing impairment may result from the disrupted coordination of cortical and subcortical neural activity, leading to increased EEG signal complexity. In this regard, Sample Entropy (SampEn) measures signal irregularity by assessing how often patterns of a given length (*m*) remain similar when extended to *m* + 1 [16]. Higher entropy values indicate greater complexity, while values near zero reflect highly predictable patterns. Entropy metrics have proven valuable in understanding various neurological disorders and aid early diagnosis, disease monitoring, and treatment evaluations. They have been applied in studies on healthy aging, Parkinson’s disease, and Alzheimer’s disease. SampEn may also provide critical insights into the neural dysfunction associated with swallowing impairments, supporting early detection and clinical assessment.

The primary objective of this study was thus to identify entropy-based EEG biomarkers for the quantitative assessment of post-stroke dysphagia to obtain novel tools for monitoring swallowing dysfunction and tracking the progress of the disease throughout neurorehabilitation.

## 2. Materials and Methods

### 2.1. Database

A 32-channel EEG was recorded from 36 post-stroke patients with dysphagia (dysphagic group), 22 post-stroke patients without dysphagia (control group), and 21 healthy elderly individuals (healthy group) at the Paré Jofré and La Pedrera hospitals in the Valencian Community, Spain. The method for diagnosing a stroke was magnetic resonance imaging, and the method used to diagnose patients with dysphagia (DG) was a volume–viscosity clinical examination (V-VST). In compliance with the Declaration of Helsinki, this study followed a protocol approved by the ethics committees of both hospitals and the Universitat Politècnica de València. The participants’ ages were 59.9 ± 6.3 years for the healthy group, 69.0 ± 12.9 years for the dysphagic group, and 68.5 ± 8.8 years for the control group, with statistically significant differences observed between groups. The proportion of female participants was 41.94% in the dysphagic group, 52.38% in the control group, and 40% in the healthy group. EEG signal acquisition was conducted on all patients on hospital admission.

### 2.2. Recording Protocol

The patients’ facial skin and mastoid areas were first cleansed with an exfoliating gel, followed by alcohol for thorough preparation. A BrainWave cap, featuring 32 electrodes arranged according to the standard 10-20 system, was then carefully positioned on the subject. The signals were captured with the TMSi SAGA 32/64+ Amplifier at a sampling frequency of 2 kHz. The TMSi user interface was implemented to check acceptable impedances up to 10 kΩ. Four disposable Ag/AgCl bipolar electrodes (MN00005 Duotrodes, Bisico, Lançon-Provence, France) were placed on the submental area with an inter-electrode distance of 21 mm for an additional recording of electromyographic (EMG) activity from the infrahyoid and suprahyoid swallowing muscles.

**Figure 1:**
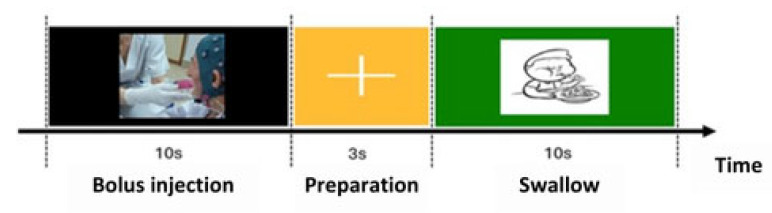
Diagram of visual instructions during swallowing protocol.

The swallowing protocol consisted of 3 stages: In the first a liquid bolus was placed in the patient’s mouth while a black screen was displayed on the computer; in the second there was a fixation cross instructing the patient to remain still. In the final stage, an image of a child appeared as a cue for the patient to initiate swallowing (swallowing marker). After verifying the patient’s safety by asking simple questions and assessing the appropriate responses, the recording protocol was manually restarted and continued until reaching a total of 40 repetitions. A summary of the test sequence and the described procedures is provided in Figure 1.

### 2.3. Preprocessing

EEG data were preprocessed with a 0.5 Hz high-pass filter and an anti-aliasing low-pass filter with a cutoff at 128 Hz and downsampled at 256 Hz. The next step involved the MATLAB FASTER (“Fully Automated Statistical Thresholding for EEG Artifact Rejection”) tool [17] to automatically eliminate defective channels. Epochs were then extracted, each comprising 3 s of the EEG signal preceding the swallowing marker (order to swallow) and 8 s afterwards. Artifact-contaminated epochs were identified by the FASTER algorithm and excluded from further analysis. To mitigate biological interference, including ocular, muscular, and cardiac artifacts, the EEG signals were band-pass-filtered between 1 and 30 Hz, re-referenced to the average of all EEG channels, and processed by Independent Component Analysis (ICA). Subsequently, non-neural components were removed using the EEGLAB toolbox in MATLAB R2024a [18] through a meticulous visual inspection of the independent components to eliminate any residual muscle activity.

### 2.4. Obtaining Biomarkers Based on Sample Entropy (SampEn)

**Figure 2:**
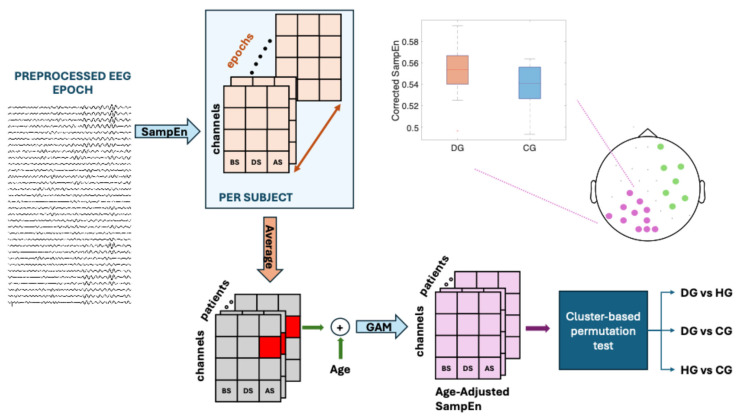
A data analysis flowchart for the theta, alpha, and beta bands. BS, DS, and AS are the swallowing states: before swallowing (BS), during swallowing (DS), and after swallowing (AS). The study populations are subjects with dysphagia (DG), controls (CG), and healthy subjects (HG). Sample Entropy (SampEn) was used to assess signal predictability, and a generalized additive model (GAM) was used to obtain age-adjusted SampEn (Corrected SampEn) by removing the influence of age. Different colors on the topographic map represent different clusters.

Figure 2 contains a flowchart outlining the process followed in this study.

**Figure 3:**
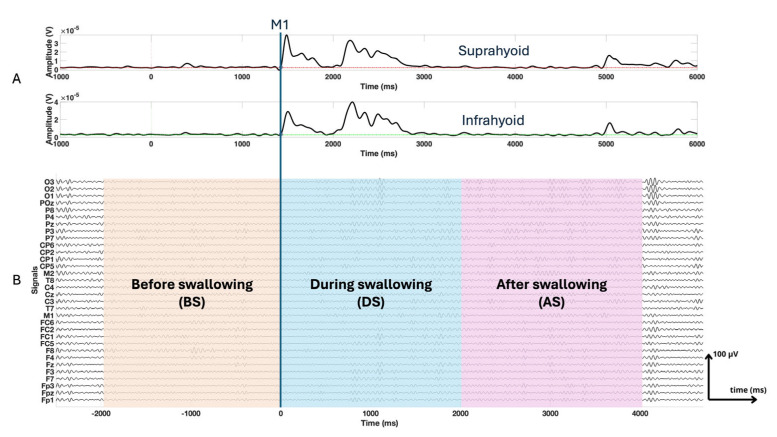
Panel (**A**) presents the electromyographic signals of the suprahyoid and infrahyoid muscles, with a temporal reference based on muscle activation during swallowing (M1). Panel (**B**) displays the windowed electroencephalography signals (BS, DS, and AS).

Fragmented swallow frequently occurred in dysphagia patients, which is characterized by multiple separate muscle activations during a single functional swallow [19,20], making precise onset and offset detection difficult. Consequently, fixed-length epochs aligned to the M1 event [21], the onset of muscle activation in the suprahyoid muscles during the swallowing process determined by EMG signals, were used to ensure consistency across all subjects. Since healthy subjects typically exhibit swallowing durations of 1 to 2 s [22], we extracted epochs extending from −2000 ms to 4000 ms, M1 being the temporal reference (0 ms) [23,24]. We further divided each epoch into 3 segments: before swallowing (BS), from −2000 ms to 0 ms prior to M1; during swallowing (DS), from 0 ms to 2000 ms; and after swallowing (AS), from 2000 ms to 4000 ms, as shown in Figure 3.

Sample entropy was used to characterize the EEG signals. This entropy measure is not only simple and easy to interpret but also well-established and widely validated in the literature. For a time series *x*[*n*], *n* = 1…Nr, SampEn was determined using the following Equation (1) [25]:

(1) $$SampEn\left(m,r\right)=-log[\frac{\sum_{i=1}^{N_{x}-m}\left[\frac{1}{N_{x}-m-1}V_{i}\left(r\right)\right]}{\sum_{i=1}^{N_{x}-m}\left[\frac{1}{N_{x}-m-1}U_{i}\left(r\right)\right]}]$$

where *Vi*(*r*) is the number of pattern pairs of length *m* + 1 similar to a pattern in the time series *x*, starting at position *i*, according to threshold *r*, which is multiplied by the standard deviation of the signal, and *Ui*(*r*) is the number of pattern pairs of length *m* similar to a pattern in the time series *x*, starting at position *i*, according to threshold *r*. These parameters count patterns *m* + 1 and m long, respectively, that meet the similarity criterion, i.e., how many of these fragments resemble each other according to a given threshold (*r*). Considering other previous studies in the literature for the EEG complexity assessment [26,27,28,29,30,31], we performed a parameter sweep over a range of SampEn hyperparameters, specifically testing embedding dimensions *m* = 2 and 3 and tolerance values *r* = 0.1, 0.15, 0.2, 0.25, and 0.3. Specifically, *m* = 3 and *r* = 0.2 were ultimately selected based on their performance stability across subjects and their physiological consistency.

Event-related desynchronization (ERD) in the alpha and beta bands is a well-known process during swallowing tasks [10,32]. Theta band activity is expected to be related to sensorimotor integration, attention, and cognitive control, all of which are crucial for coordinating voluntary and reflexive aspects of swallowing [33]. In this work, SampEn metrics were calculated for the frequency bands to characterize swallowing-related cortical dynamics: theta (4 to 8 Hz), alpha (8 to 13 Hz), and beta (13 to 30 Hz).

All the above processes were carried out using the following formula:

(2) $$SampEn_{jk}^{l}=\frac{1}{N}\sum_{p=1}^{N}\left(SampEn_{m,p}^{l}\right)$$

where *j* is the swallowing state (BS, DS, and AS), *k* corresponds to the frequency band (theta, alpha, and beta), *l* corresponds to the patient, *N* is the total number of epochs per patient, *p* is the epoch, and m is each electrode in the cortical surface.

### 2.5. Statistical Analysis

Nonparametric procedures were used to assess differences between groups because of the lack of normality in the data distribution, confirmed by the Kolmogorov–Smirnov test. The corresponding statistical test identified a significant difference in participant age between groups. Therefore, a generalized additive model (GAM) was applied to cancel out the influence of this confounding variable on the SampEn of each channel. The GAM allowed us to flexibly model the potential nonlinear influence of age on signal complexity [34]. The GAM used was as follows:

(3) $$SampEn_{ij}=\beta_{0}+s_{1}\left(Age_{i}\right)+\epsilon_{ij}$$

where *SampEn_ij_* represents the sample entropy value for subject *i* at electrode *j*, and *s*_1_(*Age*) is a smooth function (spline) that models the nonlinear effect of age. The adjusted residual, which corresponds to age-adjusted SampEn, represents the variations that cannot be explained by age.

Subsequently, a nonparametric cluster-based permutation test, as described by [35], was applied to assess statistical differences between groups. This approach effectively controls for multiple comparisons by leveraging the spatial dependence between electrodes, making it particularly well-suited for EEG data analysis. Pairwise comparisons were made between groups (DG vs. HG, DG vs. CG, and CG vs. HG).

Once significant clusters were identified (*p* < 0.05), the age-adjusted SampEn values from all electrodes within each cluster were extracted, and a mean value per subject was calculated for each cluster. These cluster-specific means were then visualized using box plots to convey both the central tendency of and variability in the data across groups. Additionally, Cliff’s delta [36] was computed as a nonparametric effect size metric, yielding a scale-independent estimate of the magnitude of the observed group differences.

## 3. Results

**Figure 4:**
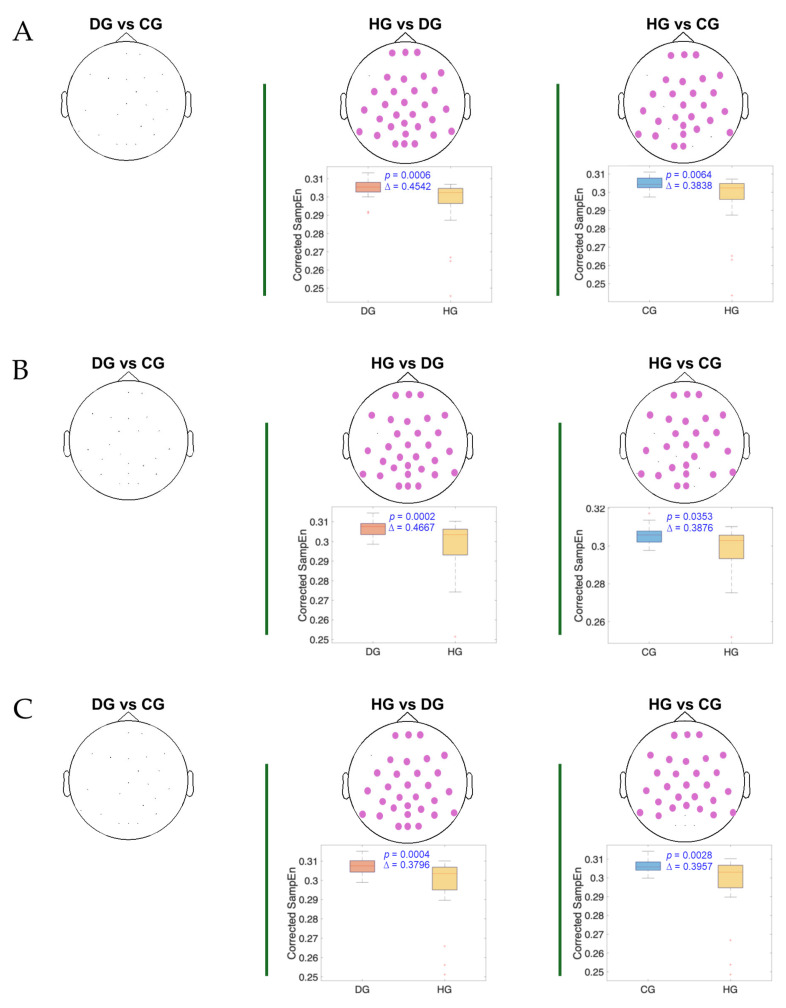
The spatial distribution of the significant cluster in the theta band, identified using a nonparametric cluster-based permutation test (parameters: *m* = 3; *r* = 0.2), across three swallowing phases: (**A**) before swallowing (BS), (**B**) during swallowing (DS), and (**C**) after swallowing (AS). Electrodes forming the significant cluster are highlighted for each group comparison: DG (dysphagic group), CG (control group), and HG (healthy group). The cluster is displayed in purple. Corresponding box plots show the distribution of the mean SampEn values within the identified cluster for each subject. The *p*-value from the cluster-based permutation test and Cliff’s delta (effect size) are reported for each comparison.

**Figure 5:**
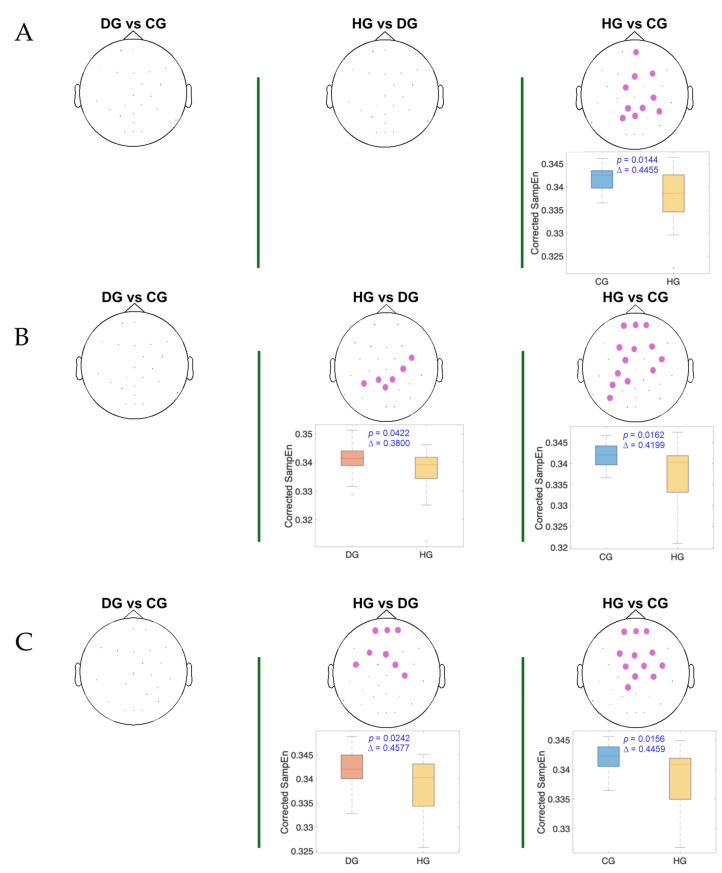
The spatial distribution of the significant cluster in the alpha band, identified using a nonparametric cluster-based permutation test (parameters: *m* = 3; *r* = 0.2), across three swallowing phases: (**A**) before swallowing (BS), (**B**) during swallowing (DS), and (**C**) after swallowing (AS). Electrodes forming the significant cluster are highlighted for each group comparison: DG (dysphagic group), CG (control group), and HG (healthy group). The cluster is displayed in purple. Corresponding box plots show the distribution of the mean SampEn values within the identified cluster for each subject. The *p*-value from the cluster-based permutation test and Cliff’s delta (effect size) are reported for each comparison.

**Figure 6:**
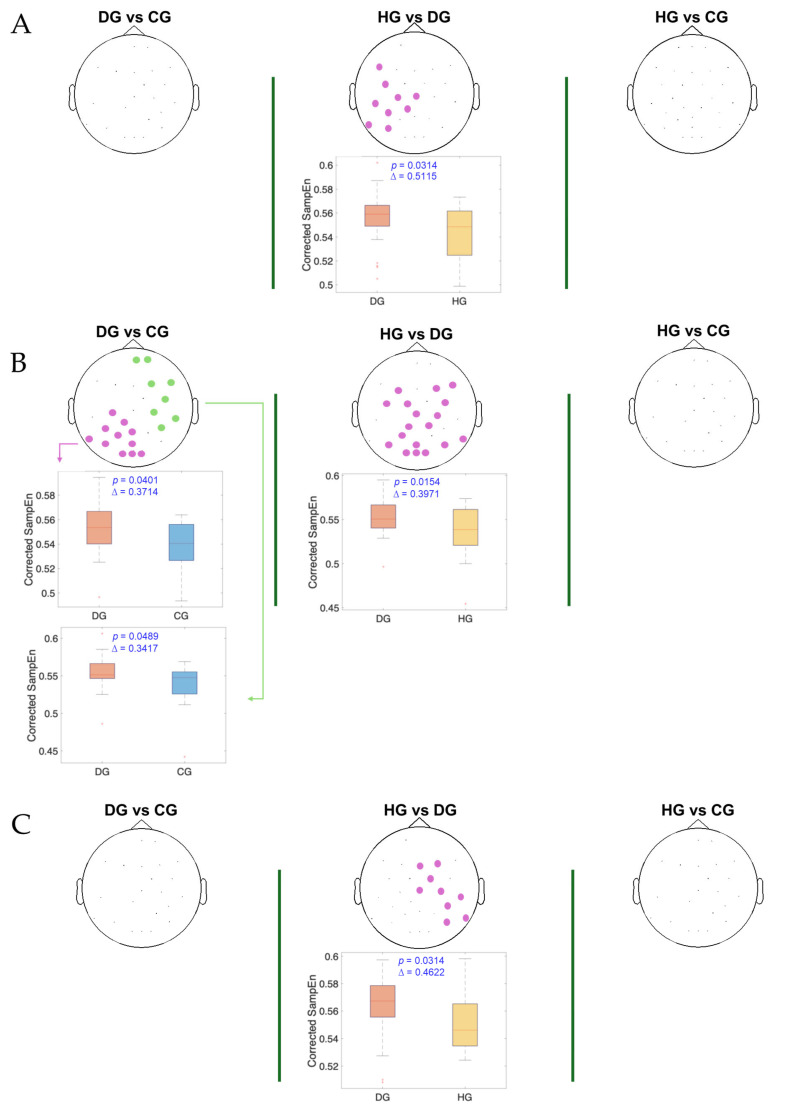
The spatial distribution of the significant clusters in the beta band, identified using a nonparametric cluster-based permutation test (parameters: *m* = 3; *r* = 0.2), across three swallowing phases: (**A**) before swallowing (BS), (**B**) during swallowing (DS), and (**C**) after swallowing (AS). Electrodes forming the significant cluster are highlighted for each group comparison: DG (dysphagic group), CG (control group), and HG (healthy group). When multiple significant clusters are detected, the first is shown in purple and the second in green. Corresponding box plots show the distribution of the mean SampEn values within the identified cluster for each subject. The *p*-value from the cluster-based permutation test and Cliff’s delta (effect size) are reported for each comparison.

Figure 4, Figure 5 and Figure 6 present the results of the statistical analysis of age-adjusted Sample Entropy (SampEn) values measured before, during, and after swallowing, across the theta, alpha, and beta frequency bands, respectively. This analysis identified spatial electrode clusters in which statistically significant differences were observed between groups in the theta, alpha, and beta frequency bands.

For both the alpha and theta bands (Figure 4 and Figure 5), notable similarities are observed, and significant clusters are observed in nearly all phases when comparing the healthy group (HG) with the other groups. The HG consistently shows lower age-adjusted SampEn values. In the theta band, the significant clusters tend to be spatially broader, involving a larger number of electrodes and covering nearly the entire cerebral cortex, while the alpha band covers mainly the central and frontocentral areas. Regarding effect sizes, higher Cliff’s delta values are observed in the theta band for the HG vs. DG comparison and in the alpha band for the HG vs. CG comparison.

For the beta band (Figure 6), distinct patterns can be observed across all swallowing phases. Significant clusters are present in the comparison between the HG and DG, with high Cliff’s delta values. A notable finding in the DS phase is the presence of two distinct clusters in the DG vs. CG comparison: one with a higher Cliff’s delta located in the left parietal and occipital regions and another with a lower effect size involving the right frontotemporal area. Additionally, across all comparisons, the distributions of age-adjusted SampEn consistently show higher values in the DG compared to the other groups.

## 4. Discussion

In this study, we conducted an evaluation using sample entropy (SampEn) metrics from EEG signals as biomarkers for the quantification and assessment of neurogenic dysphagia.

Our findings show that the theta and alpha frequency bands are particularly sensitive markers for distinguishing stroke (CG and DG) patients from the HG across all phases of the protocol (BS, DS, and AS). This pattern aligns with the existing literature identifying alterations in these bands as robust indicators of stroke-related cortical dysfunction [37]. Specifically, alpha band abnormalities were predominantly observed in the frontal and central regions, consistent with previous studies that associate alpha power reductions or disruptions in these areas with impaired cortical regulation and motor deficits following stroke [38]. The frontal and central alpha oscillations have been linked to attentional processes and motor preparation, both critical in swallowing control, which explains their particular relevance in our swallowing paradigm. In contrast, differences in the theta band extended across nearly the entire scalp, reflecting widespread cortical alterations. Elevated theta activity is a well-established marker of post-stroke cortical dysfunction, often associated with cortical slowing and reduced neural efficiency [37,39]. Importantly, during the swallowing task, stroke patients must exert greater attentional, sensorimotor, and cognitive control to compensate for impaired automatic motor function. This increased demand likely contributes to the observed global theta enhancement, reflecting both neural dysfunction and heightened task-related engagement.

It is crucial to highlight the ongoing debate concerning the role of the cerebral cortex in regulating swallowing. We found that in dysphagia patients, brain activity differed significantly from that of healthy controls in the beta band—beginning in the left hemisphere before swallowing, shifting to the central region during swallowing, and moving to the right hemisphere post-swallow. Our results support previous findings that point out that the degree of cortical involvement differs depending on the swallowing phase: the oral phase is predominantly governed by the left hemisphere, while dominance shifts during the pharyngeal phase, with the right hemisphere taking control in the esophageal phase [40,41].

The parietal neocortex plays a central role in swallowing by integrating sensory input from the oropharynx with motor functions necessary for deglutition, effectively channeling sensory signals to motor centers and coordinating activity with other motor regions [42,43]. Functional imaging studies in healthy individuals have shown that the motor planning and execution of voluntary swallowing primarily involve the lateral pericentral cortex, frontoparietal operculum, and specifically the left postcentral gyrus when tongue movement is excluded [44]. Furthermore, neuroimaging and electrophysiological studies report significant beta band event-related desynchronization (ERD) in the inferior precentral gyrus and left parietal cortex during swallowing, indicating cortical activation [10,32]. Notably, our preliminary data also confirmed ERD in this area, highlighting its relevance. Our findings showed that dysphagic patients exhibit reduced EEG signal organization (i.e., higher Sample Entropy values) in the left parietal region—an area crucial for monitoring and executing swallowing—suggesting disruption in the neural mechanisms governing these processes. The absence of or reduction in ERD and increased entropy may reflect disorganized neural activity and impaired motor coordination during swallowing [45]. This is consistent with prior studies identifying the left postcentral gyrus and Sylvian fissure as key regions in swallowing control [40,41]. These findings support the potential of beta band Sample Entropy, particularly in the left parietal and motor cortex, as a biomarker for detecting dysphagia, in agreement with prior evidence [10,32].

Interestingly, our results revealed significant differences in SampEn values in the right frontotemporal region between the DG and the other groups in the beta band. This finding suggests a possible role for this cortical area in the altered neuronal processing associated with swallowing dysfunction. Furthermore, functional imaging studies have shown that these regions become more active in post-stroke patients with dysphagia, possibly reflecting a reorganization of the cortical swallowing network to recruit additional resources for motor planning and execution [46].

Swallowing has also been associated with increased gamma activity in the Sylvian fissure, as demonstrated by intracranial EEG studies [32]. In this work, we did not assess the alteration in the gamma band in dysphagia patients. This is mainly motivated by the fact that due to the relatively low signal-to-noise ratio, gamma activity remains challenging to detect in scalp electrodes.

ERD-based metrics limit the ability to capture more complex aspects of brain dynamics, such as temporal variability or the complexity of the organization of neural activity, whereas entropy metrics offer a complementary perspective by quantifying the degree of disorder, unpredictability, or complexity of the EEG signal. The integrated use of power and entropy metrics not only enhances the interpretation of neuronal activity changes but also provides a more comprehensive understanding of the mechanisms underlying cognitive and pathological processes, which in turn increase the physiological significance of the findings derived from EEG signals.

Our findings reinforce the hypothesis that dysfunction in cortical modulation in dysphagic subjects could be related to altered neuronal connectivity, affecting both the planning and execution of the swallowing motor act. In addition, the beta band, known for its role in regulating motor and cognitive tasks [47,48,49,50], could be a sensitive marker of functional changes in dysphagia-related cortical activity. This study on entropy proposes an effective biomarker for distinguishing dysphagic patients, facilitating a more precise characterization of the disorder and informing of the development of targeted therapeutic approaches. SampEn assessment during swallowing has the potential to be established as a crucial tool for identifying early abnormalities and formulating specialized rehabilitation strategies for individuals with dysphagia.

This study has several limitations. First, our relatively small sample may underestimate the discriminatory power of SampEn, and a larger cohort would not only strengthen statistical robustness but also allow for the exploration of more subtle group differences. Future work should recruit diverse, larger patient populations to validate and refine SampEn for clinical use. Second, although we incorporated Cliff’s delta to gauge effect size independently of sample size and distribution assumptions, we did not correlate EEG biomarkers with dysphagia severity. Longitudinal studies that track patients before and after specific therapeutic interventions—using both SampEn and complementary complexity measures—could elucidate how these metrics evolve with disease progression and recovery, thereby establishing their prognostic value. Third, the high inter-subject variability inherent to our cross-sectional design highlights the need for repeated measures within individuals over time. Such designs would clarify within-subject changes and help disentangle trait versus state effects in neurogenic dysphagia. Finally, integrating SampEn with other EEG features (e.g., spectral power, connectivity), peripheral physiological metrics, and clinical scales in multimodal predictive models represents a promising avenue for enhancing diagnostic precision and tailoring personalized rehabilitation strategies. By addressing these points, future research can move beyond proof of concept toward robust, clinically actionable biomarkers for dysphagia.

Despite these limitations, the present work paves the way towards an objective and quantitative assessment of neurogenic dysphagia using the non-invasive and cost-effective EEG technique. It could have an impact on social and health care that would decongest health care centers and lead to an improvement in dysphagia patients’ quality of life, as neurorehabilitative and pharmacological treatments could be personalized. On the other hand, a range of research is now open for quantifying dysphagia using other entropy metrics or frequency-based entropies to obtain complementary biomarkers to those provided by SampEn.

## 5. Conclusions

Post-stroke patients—regardless of dysphagia status—exhibited elevated age-adjusted SampEn across multiple cortical regions, with particularly widespread pronounced increases in the theta band and the frontal–central alpha band, before, during, and after swallowing, relative to healthy controls. These findings point to a generalized disruption of neural dynamics following stroke, likely stemming from impaired sensorimotor integration and compensatory cognitive effort during swallowing. Crucially, further analysis revealed that beta band dysphagia patients exhibited significantly higher values in age-adjusted SampEn in the right frontotemporal and left parietal cortices than non-dysphagic stroke patients. The lack of reduced entropy during swallowing, particularly in the left parietal cortex, may reflect cortical discoordination and impaired sensorimotor processing associated with swallowing dysfunction, serving as a potential neurophysiological marker of dysphagia.

Quantifying these alterations provides potential objective and quantitative biomarkers for diagnosing and assessing neurogenic dysphagia, using EEG as a non-invasive and cost-effective technique.

## Data Availability

The data supporting the findings of this study are not publicly available due to privacy and ethical restrictions, as they contain personal and sensitive information. Data can be made available upon reasonable request to the corresponding author, subject to approval by the relevant ethics committee and compliance with data protection regulations.
